# Implementation of medication review type 2a in community pharmacies: a longitudinal survey study

**DOI:** 10.3389/fphar.2025.1685922

**Published:** 2025-11-12

**Authors:** Eline Tobback, Isabelle De Wulf, Ella Wauters, Delia Mendez Santos, Stéphanie Patris, Olivia Dalleur, Eline Tommelein, Veerle Foulon, Geneviève Philippe, Carine De Vriese, Stephane Steurbaut, Nathalie Duquet, Guido R. Y. De Meyer, Hans De Loof

**Affiliations:** 1 Laboratory of Physiopharmacology, University of Antwerp (UA), Antwerp, Belgium; 2 Association of Pharmacists Belgium (APB), Antwerp, Belgium; 3 Clinical Pharmacy Unit, Faculty of Medicine, Pharmacy and Biomedical Sciences, University of Mons (UMONS), Mons, Belgium; 4 Clinical Pharmacy and Pharmacoepidemiology Research Group, Louvain Drug Research Institute, UCLouvain, Brussels, Belgium; 5 Pharmacy Department, Cliniques Universitaires Saint-Luc, UCLouvain, Brussels, Belgium; 6 Department of Pharmacological and Pharmaceutical Sciences, Research Group Experimental Pharmacology, Vrije Universiteit Brussel, Jette, Belgium; 7 Department of Pharmaceutical and Pharmacological Sciences, Clinical Pharmacology and Pharmacotherapy, KU Leuven, Leuven, Belgium; 8 Center for Interdisciplinary Research on Medicines (CIRM)/Pharmacy Practice Research Group, University of Liège, Liège, Belgium; 9 Service de Pharmacologie, Pharmacothérapie et Suivi Pharmaceutique, Université Libre de Bruxelles, Brussels, Belgium; 10 Pharmacy Department, Universitair Ziekenhuis Brussel (UZ Brussel), Jette, Belgium; 11 Vitality Research Group, Vrije Universiteit Brussel, Jette, Belgium; 12 Infla-Med Research Center of Excellence, University of Antwerp, Antwerp, Belgium

**Keywords:** pharmaceutical services, medication therapy management, community pharmacy services, health services accessibility, interprofessional relations, surveys and questionnaires, health policy

## Abstract

**Introduction:**

Medication Review Type 2a (MR2a), introduced to enhance medication use and health outcomes, has been reimbursed in Belgian community pharmacies since April 2023; however, its implementation status remains unreported.

**Aim:**

This longitudinal survey study aimed to document MR2a implementation among community pharmacists by identifying both facilitating and hindering factors.

**Methods:**

A bilingual online survey was distributed quarterly from October 2023 to October 2024 to all Belgian community pharmacies. The 45-item questionnaire—featuring multiple-choice, Likert scale, and open-ended questions—captured pharmacists’ experiences about MR2a.

**Results:**

Across all rounds (*n* = 708), consistent findings emerged regarding key barriers and facilitators to MR2a implementation. Major challenges included time limitations, privacy-compliant communication systems, personnel shortages, inadequate consultation spaces and poor collaboration with general practitioners. Training and patient engagement were identified as key facilitators, yet ongoing stakeholder support was considered essential to ensure successful MR2a implementation.

**Conclusion:**

Repeated surveys offer timely insights into MR2a implementation in community pharmacies. The slow progress confirms existing obstacles and stresses the importance of continued support and improved interprofessional collaborations. These insights are expected to inform MR implementation locally and internationally.

## 1 Introduction

By 2030, one in six people is expected to be aged 60 years or older, reflecting a global rise in life expectancy, a trend observed across many countries, including Belgium (WHO, 2024; StatBel, 2024). Aging populations face higher chronic disease rates, often requiring multiple medications (polypharmacy), which raises the risk of drug-related problems (DRPs) and complicates therapeutic management (Chen, 2016; Chiaranai et al., 2018; Hajjar et al., 2007; Masnoon et al., 2017; UpToDate, 2024; Kim and Parish, 2017). Medication Review (MR) can address these complexities (Wang et al., 2025). According to the Pharmaceutical Care Network Europe (PCNE), MRs are structured evaluations of a patient’s medications to optimize medication use and improve outcomes by identifying and addressing DRPs (Griese-Mammen et al., 2018). The PCNE classifies MRs into three types: a simple form (Type 1), an intermediate form (Type 2) and a comprehensive form (Type 3) (Griese-Mammen et al., 2018).

MR is already implemented in many European countries (Imfeld-Isenegger et al., 2020; Bulajeva et al., 2014; Brandt et al., 2020). In Belgium, Medication Review Type 2a (MR2a), as defined by the PCNE classification, has only recently been adopted (Griese-Mammen et al., 2018; KAVA et al.). Between December 2016 and May 2017, a pilot study, called the SIMENON project, was launched by the Association of Pharmacists Belgium (APB) in collaboration with local pharmacist associations and research teams from three Belgian universities (Wuyts, 2019; Lelubre et al., 2019). This project aimed to assess the feasibility of MR2a in Belgian pharmacies, and study the challenges associated with its implementation. Several key barriers were identified: patient refusal, time constraints, staff shortages, organizational challenges, and limited awareness among general practitioners (GPs) and patients. Facilitating factors included team size, motivation, regular team meetings, and GP collaboration, with pharmacists working alone demonstrating feasibility through organizational adjustments (Wuyts, 2019; Lelubre et al., 2019). Later, in 2017–2018, the Royal Pharmacists Association of Antwerp (KAVA) initiated a pilot project to implement the more advanced MR3 (Robberechts, 2024; KAVA, 2025).

Since April 2023, MR2a is a reimbursed pharmacist service in Belgium for patients utilizing five or more reimbursed chronic medications (BCFI, 2023). MR2a can be initiated by the pharmacist, GP, or at the patient’s request, where the first option is the predominant scenario. By reviewing the patient’s medication history and conducting a structured interview with the patient, MR2a assesses adherence, identifies DRPs, explores patient concerns and provides clarification or education where needed. This patient-centered approach enables the pharmacist to support safe and effective medication use and, if necessary, initiate follow-up with other healthcare providers (Griese-Mammen et al., 2018; KAVA et al.; RIZIV, 2024a). To support the implementation of this service, the national pharmaceutical association (APB) provided various resources, including a structured protocol outlining seven steps from service initiation to medication therapy plan updates (APB, 2025a). Additional tools, such as informational materials, a guide for the identification of DRPs and an instructional manual for the electronic form (e-form) were also provided. The e-form was designed to structure and standardize medication reviews and to report findings needed for reimbursement of the service (APB, 2025a; FarmaFlux, 2023). Additionally, the GheOP^3^S tool is fully integrated with the e-form for individuals aged 65 and over (Ghent, 2023).

Already since 2019, various training programs, including e-learning, webinars, and workshops, have been offered by both the Flemish *Institute for Permanent Study for Pharmacists* (IPSA) and the Wallonian *Société Scientifique des Pharmaciens Francophones* (SSPF) to prepare pharmacists for the implementation of MR2a, once it would be reimbursed. Basic training sessions introduced the protocol and included case-based practice using the e-form, while advanced courses provided in-depth discussions on specific diseases, clinical guidelines, and laboratory data interpretation (IPSA, 2025; SSPF, 2025). Furthermore, all universities have incorporated MRs training into their curricula and the majority now require students to perform MRs during their internships.

As already exemplified by the 2016–2017 APB pilot study on MR2a, the implementation of innovative healthcare services often presents both challenges and enabling factors (Lelubre et al., 2019). This study seeks to shed light on the current state of the service’s countrywide implementation in Belgium, evaluating whether progress has been achieved and if challenges persist. This research is anticipated to inform further MR implementation efforts at both national and global levels.

Through a repeated online survey, this study aimed to examine whether observable differences exist over time to gain insight into the evolution and implementation willingness of MR2a in Belgium. Through this questionnaire, pharmacists were asked about their experiences with this service to gain insights into potential barriers as well as facilitating factors in the implementation of MR2a.

## 2 Materials and methods

### 2.1 Study design and setting

This survey was carried out among pharmacists in Belgium to explore both the current implementation and the willingness to adopt the MR2a service in community pharmacy settings. A longitudinal design was used to capture the evolution of MR2a over time. The survey was distributed to Belgian community pharmacists at 4 fixed points between October 2023 and October 2024. This design allowed the exploration of pharmacist’s evolving experiences and perceptions. The study obtained ethical approval from the Local Biomedical Ethics of the University hospital of Antwerp Committee (ref nr ID5629, 21 August 2023).

### 2.2 Participants

Inclusion criteria for participation were minimal: respondents needed to be pharmacists employed in a Belgian community pharmacy. This broad inclusion approach was chosen to receive a comprehensive view of both willingness to implement and actual progression of MR2a implementation across diverse community pharmacy settings. No demographic data such as the participants’ gender, age or specific role within the community pharmacy were gathered, in order to retain focus on implementation-related factors. Only the time since graduation, categorized as less or more than 5 years ago, was collected as background characteristic.

### 2.3 Questionnaire development and variables

An initial draft of the questionnaire (in Dutch) was created in June 2023 through collaboration between researchers at the University of Antwerp and the national pharmaceutical association (APB) and was subsequently refined through multiple rounds of revision (HDL, IDW, ND, GDM). In July 2023, this interim version was discussed and approved at a physical meeting with representatives of most Belgian Universities and representatives of local professional organizations in the *Center of Scientific Development for Pharmacists* (CWOA) of the national pharmacist association (APB). Final adjustments aimed to limit completion time to 10 min. This final version was carefully translated into French by ND to obtain a bilingual survey. The Dutch-to-French translation was executed by N. D., a professional routinely responsible for accurate translations of key documents for the Belgian national pharmacists’ association, APB. Survey validation was achieved through collective discussion among all authors, several of whom engage in both academic research and part-time practice within the community pharmacy settings.

**FIGURE 1:**
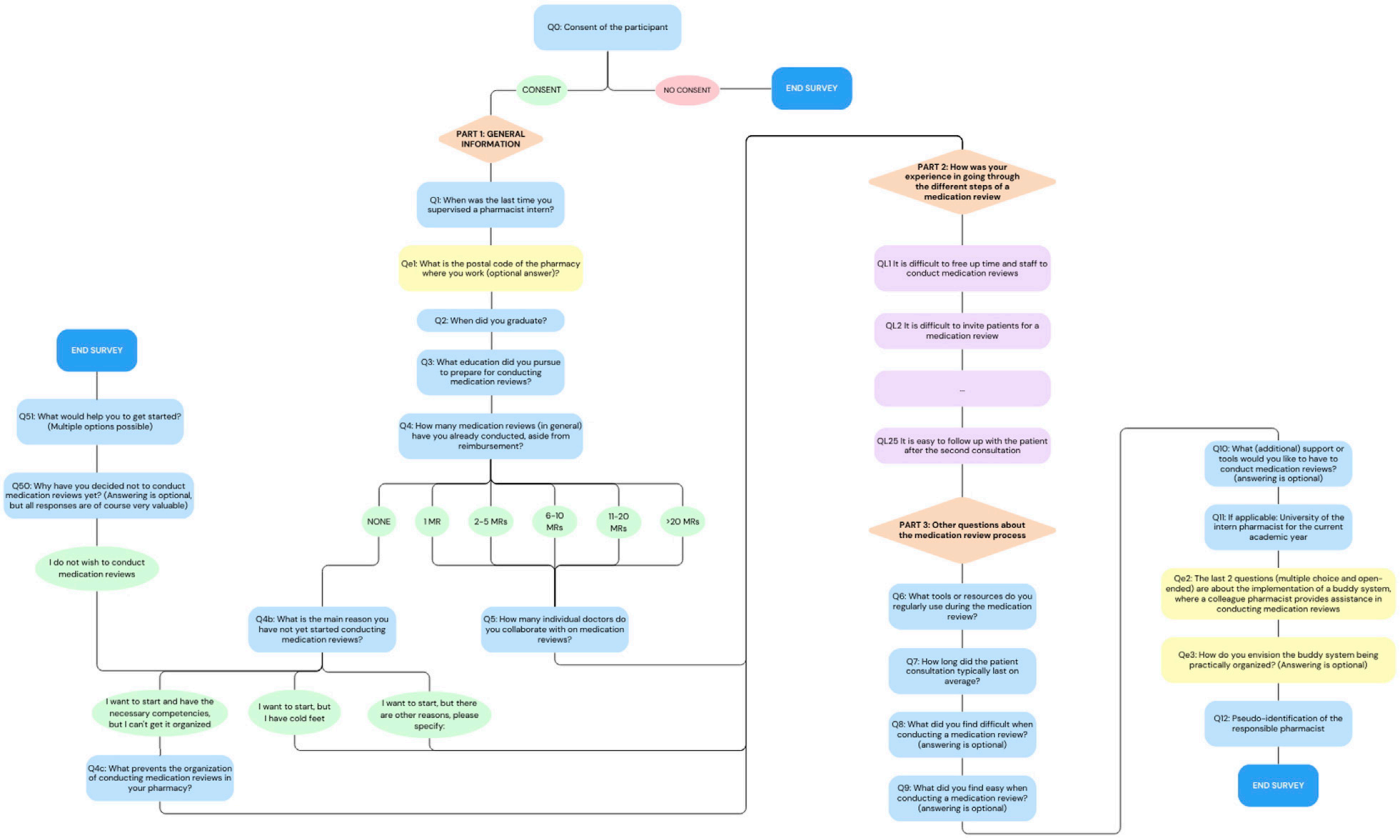
Flowchart of the online survey. Orange triangles represent the different sections of the survey, blue rectangles indicate the multiple-choice and open-ended questions, purple rectangles show Likert Scale questions, ovals indicate answer option and yellow rectangles highlight questions that were added later in the process (For the full questionnaire, see in the appendix).

The final questionnaire had three parts: (i) General information, (ii) Statements about the experiences with the MR2a process for which the level of agreement was measured using a 5-point Likert Scale (Joshi et al., 2015): Strongly agree, agree, neutral, disagree, strongly disagree or not applicable and (iii) Other questions about the MR2a process, with a few open-ended questions that allowed participants to express their opinions in more detail. Participants were asked to enter a unique code, known only to them, to anonymously track their responses across different survey rounds. The full questionnaire (translated in English) can be found in the appendix. Since the presentation of some questions depended on the participants’ previous answer, Figure 1 provides an overview of the survey flow.

### 2.4 Data collection

The survey was conducted anonymously using Qualtrics® (2005). The survey was scheduled every 4 months, with the initial round taking place from late September to early October 2023, followed by the next round in February 2024. Following the second round, potential modifications to the survey for subsequent rounds were considered. Ultimately, only two items were added: (i) a question about the pharmacy’s postal code, allowing for the examination of regional differences in participation and opinion and (ii) a question about the willingness to participate in a buddy system. The next two rounds took place in June 2024 and October 2024.

The dissemination of the survey was aided by national and many regional pharmacists’ organizations through their regular communication channels. Invitations emphasized that all pharmacists were welcome to participate in the survey, regardless of whether they had already performed a MR2a or regardless their function obtained in the pharmacy. Students from various universities were encouraged to motivate the pharmacists overseeing their internship to complete the survey. This invitation was repeated during dedicated preceptors evenings organized by the universities. In the second round, the questionnaire was also shared via social media in pharmacist forums. During the fourth round, pharmacies also received direct email invitations to take part in the survey. Participants were asked to complete the survey only once per pharmacy although this could not be verified due to the anonymous nature of the survey.

### 2.5 Data analysis

Any questionnaire that was more than halfway completed was included in the data analysis to ensure the retention of relevant information. The data analysis and visualization was conducted using Microsoft Excel version 16.98 (Microsoft). The statistical analyses were performed using the Kruskal–Wallis test in IBM SPSS software (IBM, 2025). A significance level of p = 0.05 was applied throughout the analyses.

For the qualitative data from open-ended questions, all responses were carefully reviewed and subsequently categorized into several predefined thematic categories. These thematic categories were defined through internal discussion between ET, HDL and GDM, based on the most frequently recurring topics in the open-ended responses. The actual categorization of responses was primarily guided by the presence of relevant key words associated with each theme. Since open responses may address multiple themes, some were allocated to more than one category, which may cause overall percentages to exceed 100%.

## 3 Results

The dataset of this study is available in the [figshare] repository with the identifier DOI: 10.6084/m9.figshare.29617805.

### 3.1 Participation rate and respondents’ language preference

A total of 954 pharmacists consented to participate, with 210 in round 1 (R1), 305 in round 2 (R2), 226 in round 3 (R3) and 213 in round 4 (R4). To maximize data inclusion, all surveys completed at least 50% were retained for analysis, resulting in 150 participants in R1, and 237, 174 and 147 in R2, R3 and R4, respectively. Table 1 provides an overview of the consenting participants and corresponding completion rates.

**TABLE 1:** Summary of consent and completion rates per survey round.

Survey completion level	R1	R2	R3	R4	Total
Consenting participants	210	305	226	213	954
Participants completing >20% of the survey	160	253	191	164	768
Participants completing >50% of the survey	150	237	174	147	708
Participants completing 100% of the survey	129	214	128	109	580

Due to the small number of participants that opted to consistently input a private key, we did not further analyse the individual trajectories of participants.

Language preferences were evenly split between Dutch and French in the first two rounds, with a slight shift towards Dutch in later rounds. A summerized overview of language preferences data is provided in the Supplementary Material.

### 3.2 Current adoption and future willingness to implement MR2a

**FIGURE 2:**
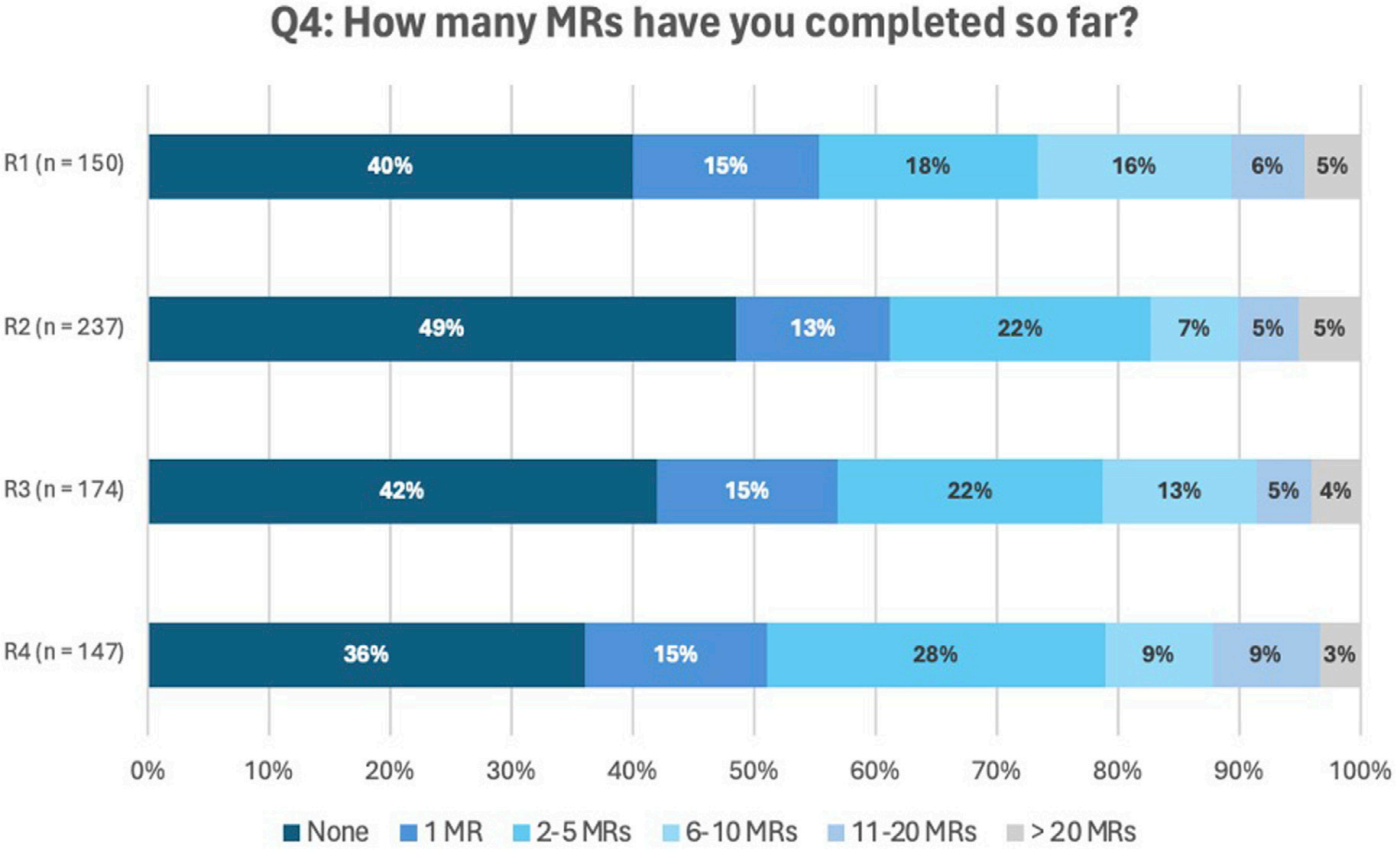
Answers to question Q4: *How many MRs have you completed so far* *?*

Figure 2 represents the number of MR2as performed over the different rounds by the participating pharmacists (results question Q4). Between 36% and 49% of the participants reported having performed none, while the majority indicated having carried out at least one review, most frequently ranging from one to five performed MR2a. A small but consistent proportion (3%–5%) stated they had conducted more than 20 MRs.

Participants who reported performing over 20 MR2as were asked to specify the exact number. These specific responses are provided in the Supplementary Material.

Pharmacists who indicated they had not yet performed a MR most often cited ‘organizational difficulties’ and reasons classified as ‘Others.’ The most frequently cited reasons were difficulties with organization and the category ‘Others.’ Within the latter category, the main barriers were lack of time (35%), absence of educational training (19%), challenges in collaboration with GPs (15%) and staff shortages (10%). An overview off all responses per round can be found in the Supplementary Material.

### 3.3 Education and training initiatives on medication review

**FIGURE 3:**
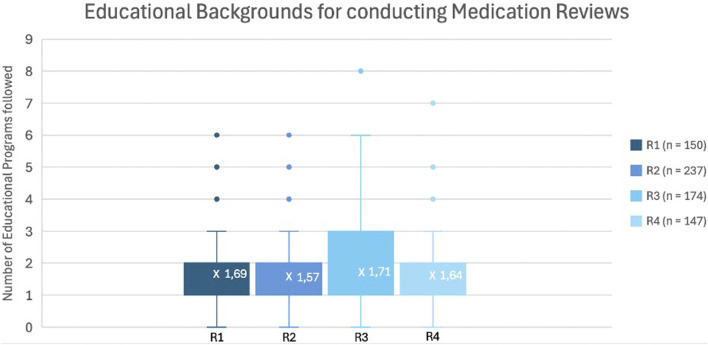
Box-and-Whisker plot representing the number of completed training programs about MR across the different rounds.

Given the importance of training in the implementation of a new service, the number of MR training sessions previously attended was assessed and is shown in Figure 3. The median number of training sessions attended remained relatively stable across the survey rounds, ranging between 1,6 and 1,7 per participant.

**FIGURE 4:**
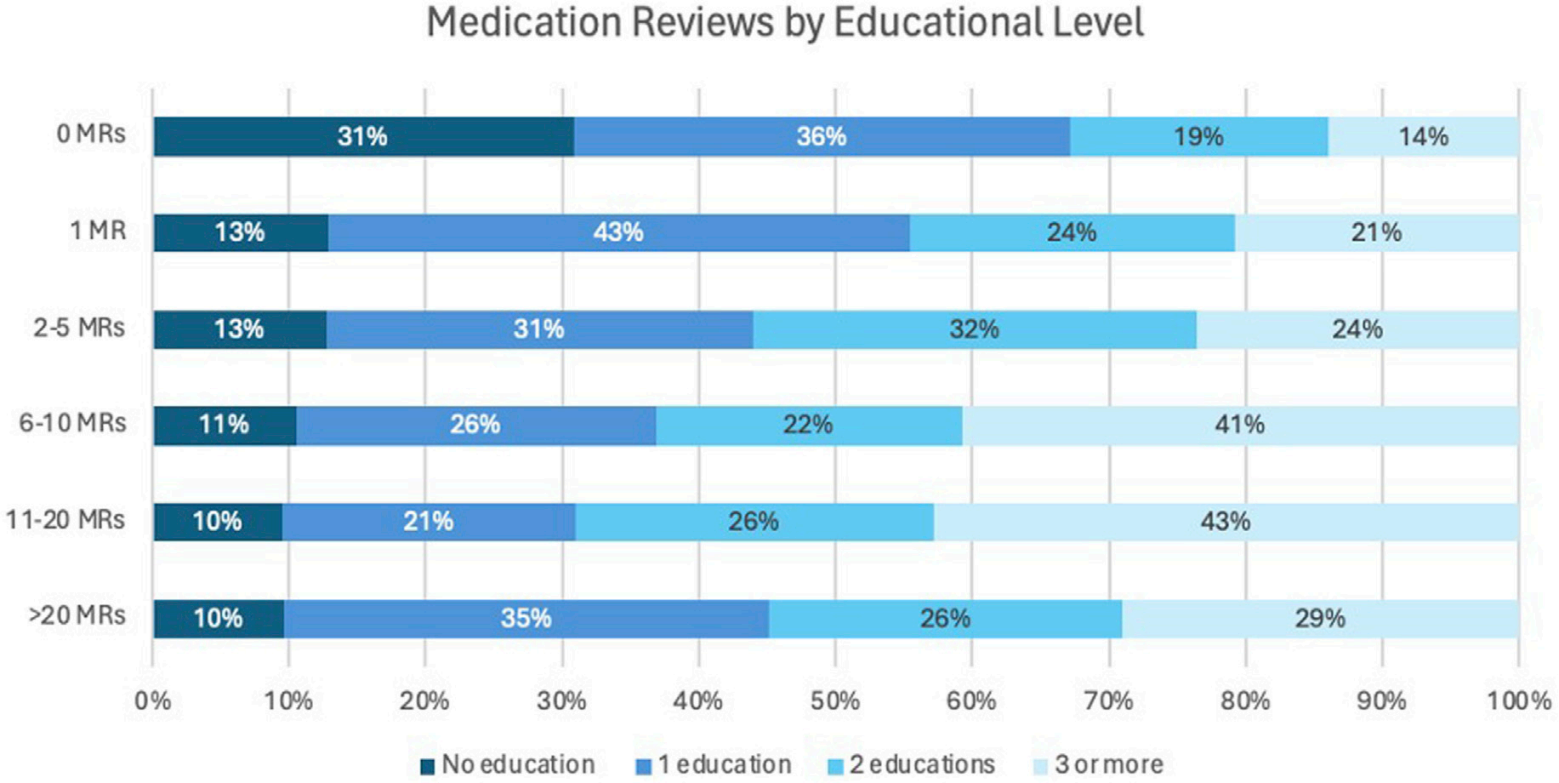
Number of MRs in relation to the number of training programs completed.

In addition, the relationship between the number of MR2a and the number of training sessions was examined in Figure 4. Among participants who had not performed an MR, 31% had not taken up specific training, while the remaining 69% had completed at least one MR training. Conversely, over 85% of participants who had performed MR2a interventions participated in at least one training, while a small subset (7%) conducted MR2as without formal training.

### 3.4 Data about the in-practice implementation

#### 3.4.1 The duration of the patient consultation

**FIGURE 5:**
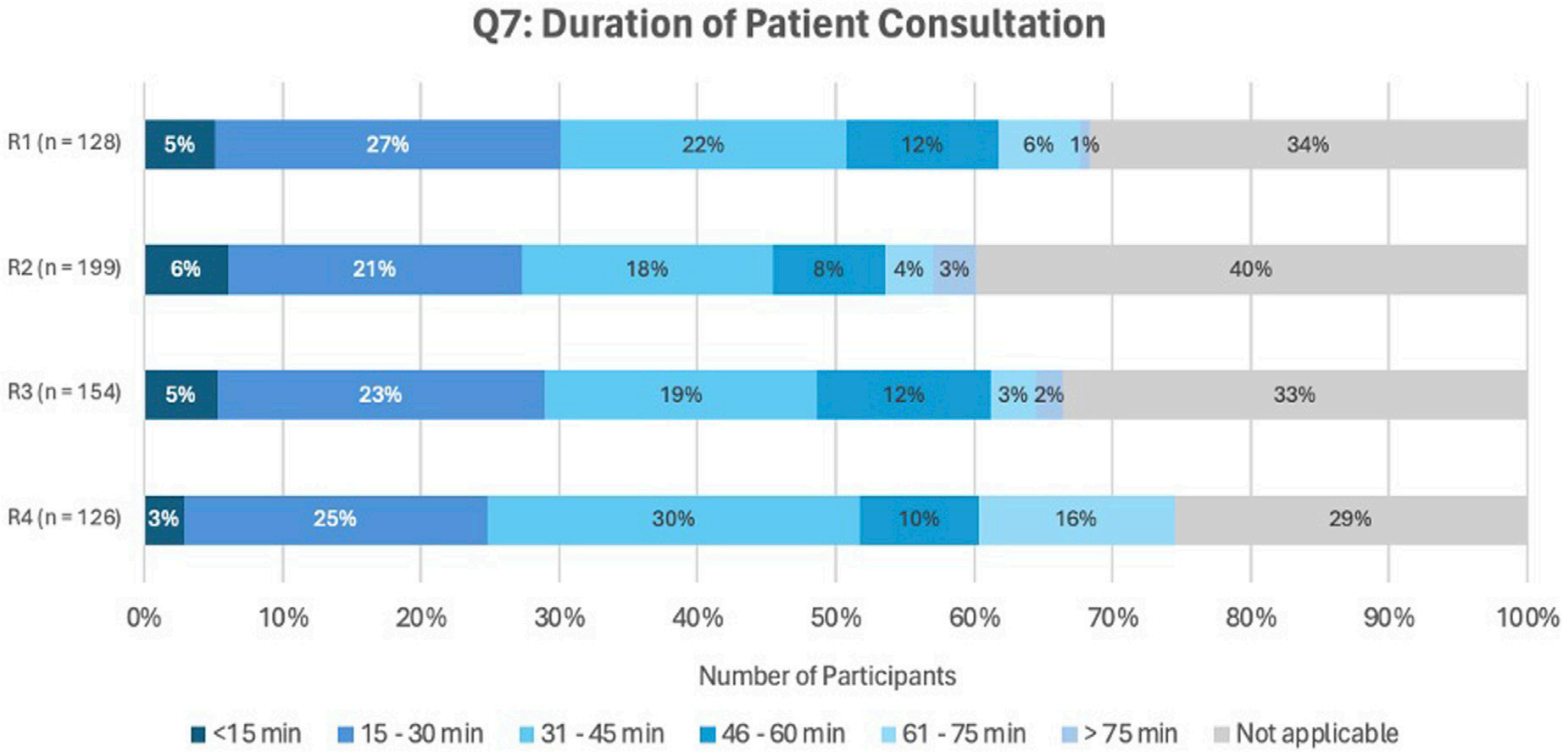
Answers to question Q7, asking about the duration of the patient consultation.

Figure 5 shows the distribution of patient consultation durations across all rounds (answers to question Q7). The majority reported a common consultation time of 15–60 min.

#### 3.4.2 Sources and tools used during a MR2a

**FIGURE 6:**
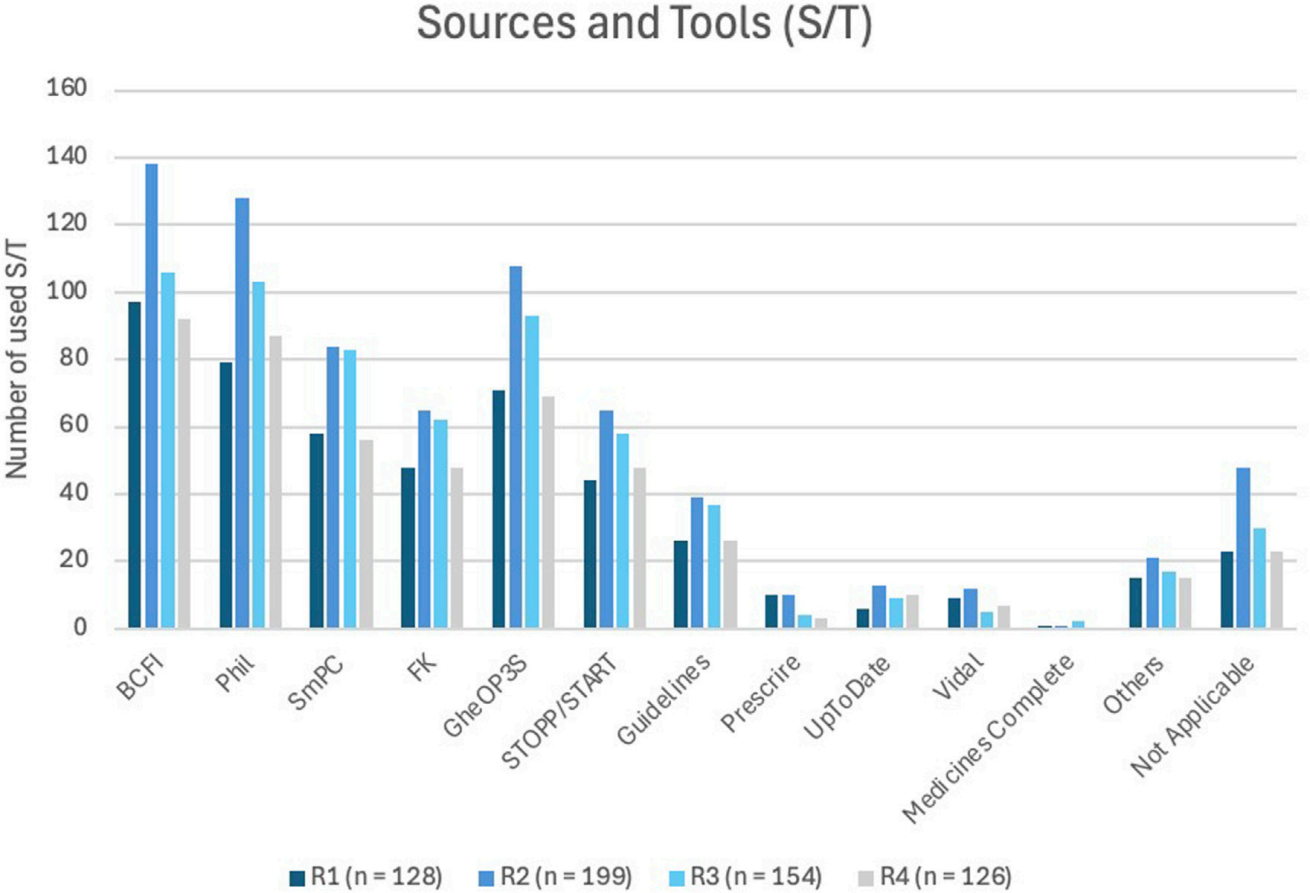
Most commonly used sources and/or tools (S/T) reported across all survey rounds. BCFI (Belgian Center for Pharmacotherapeutic Information) (BCFI, 2024); Phil (Pharmaceutical Information System) (APB, 2023); SmPC (Summary of Product Characteristics) (EMA, 2025); FK (Pharmacotherapeutic compass) (ZorginstituutNederland, 2025); GheOP3S (Ghent Older People’s Prescriptions community Pharmacy Screening) (Ghent, 2023); STOPP/START (Screening Tool of Older Persons’ Prescriptions/Screening Tool to Alert to Right Treatment) (Gallagher et al., 2008); Guidelines: Domus Medica (DomusMedica, 2025), NHG-richtlijnen (NHG, 2025), SSMG(SSMG, 2025); Prescrire^®^ (Prescrire, 2025); UpToDate^®^ (Kluwer); Vidal^®^ (Vidal, 2025); MedicinesComplete^®^ (RoyalPharmaceuticalSociety, 2025).

Figure 6 illustrates the sources pharmacists utilize during a MR2a. The most frequently used resources were the BCFI website (Belgian Centre for Pharmacotherapeutic Information) (BCFI, 2024), the online Phil database (PHarmaceutical Information Library) (APB, 2023) and the Summary of Product Characteristics (SmPC) (EMA, 2025). Furthermore, the most commonly used tools were GheOP^3^S-tool and the STOPP/START criteria (Ghent, 2023; Gallagher et al., 2008; O'Mahony et al., 2023; O'Mahony et al., 2015).

**FIGURE 7:**
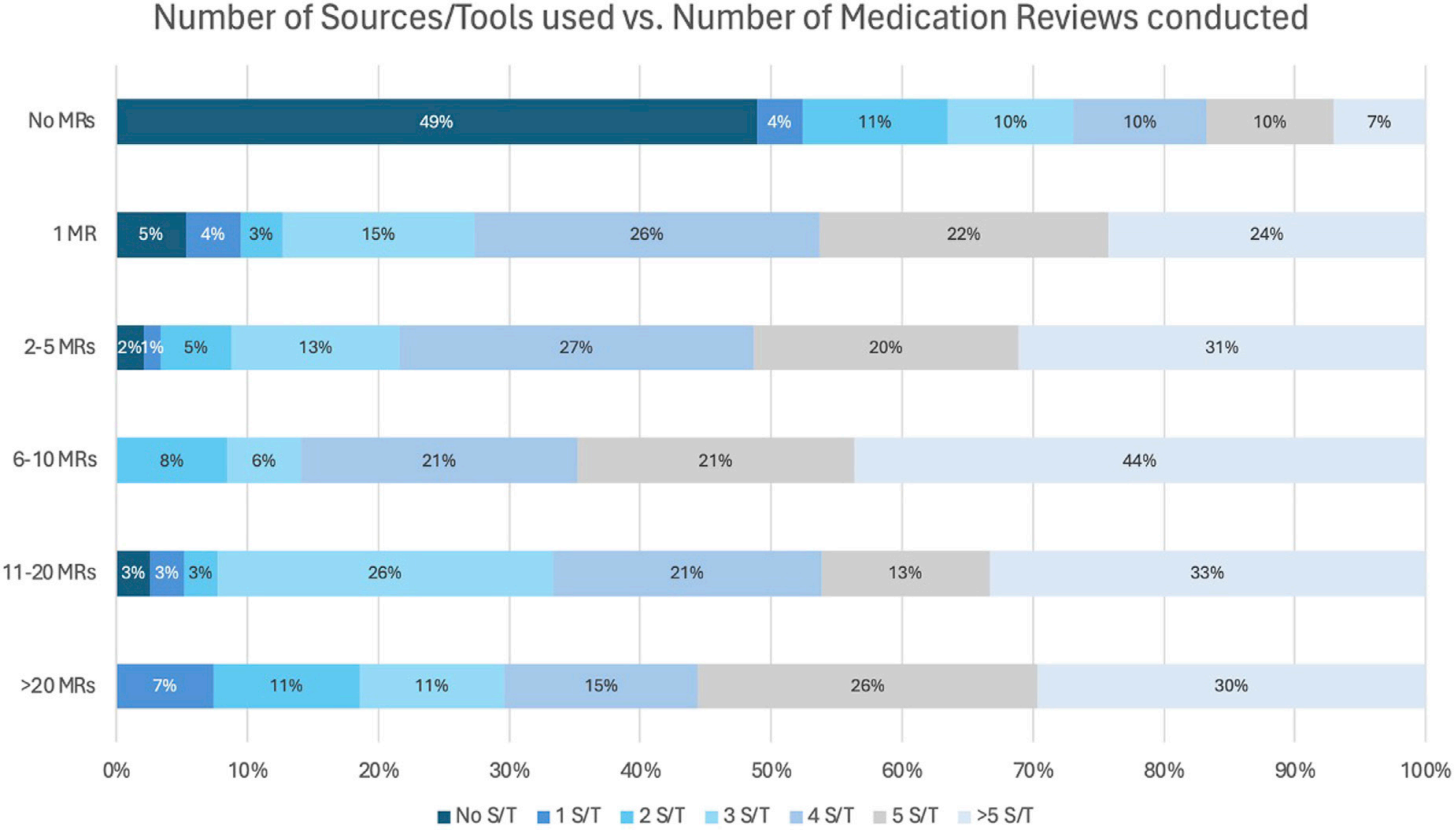
Number of sources/tools (S/T) used during a MR in relation to the number of performed MR2a.

Additionally, the number of sources and tools (S/T) used was compared to the number of MRs performed. This is displayed in Figure 7. Among participants performing MR2a, most used at least 1 S/T, with over 70% employing four or more, while only a small minority (0%–5%) reported conducting MR2a without any reference materials.

#### 3.4.3 Likert Scale questions about the experiences with MR2a

**FIGURE 8:**
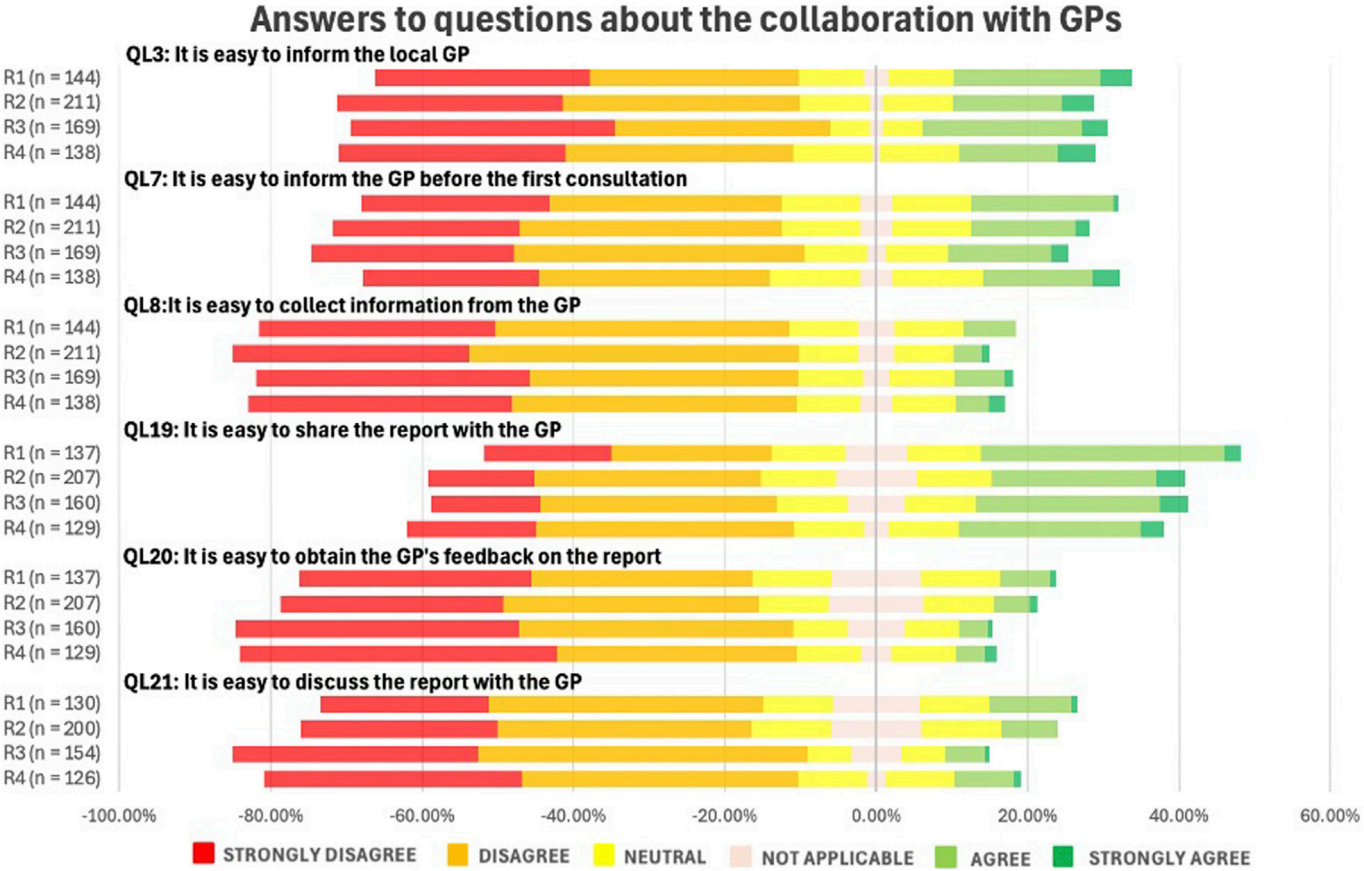
Likert Scale questions (QLs) about the pharmacists’ perception on collaboration with the general practitioners (GPs) (See in the appendix for full questionnaire).

Figure 8 illustrates pharmacists’ perceptions of collaboration with GPs. Overall, this collaboration was perceived as challenging. No statistically significant differences were observed across rounds (p-value = 0.989), indicating that responses remained stable over time.

**FIGURE 9:**
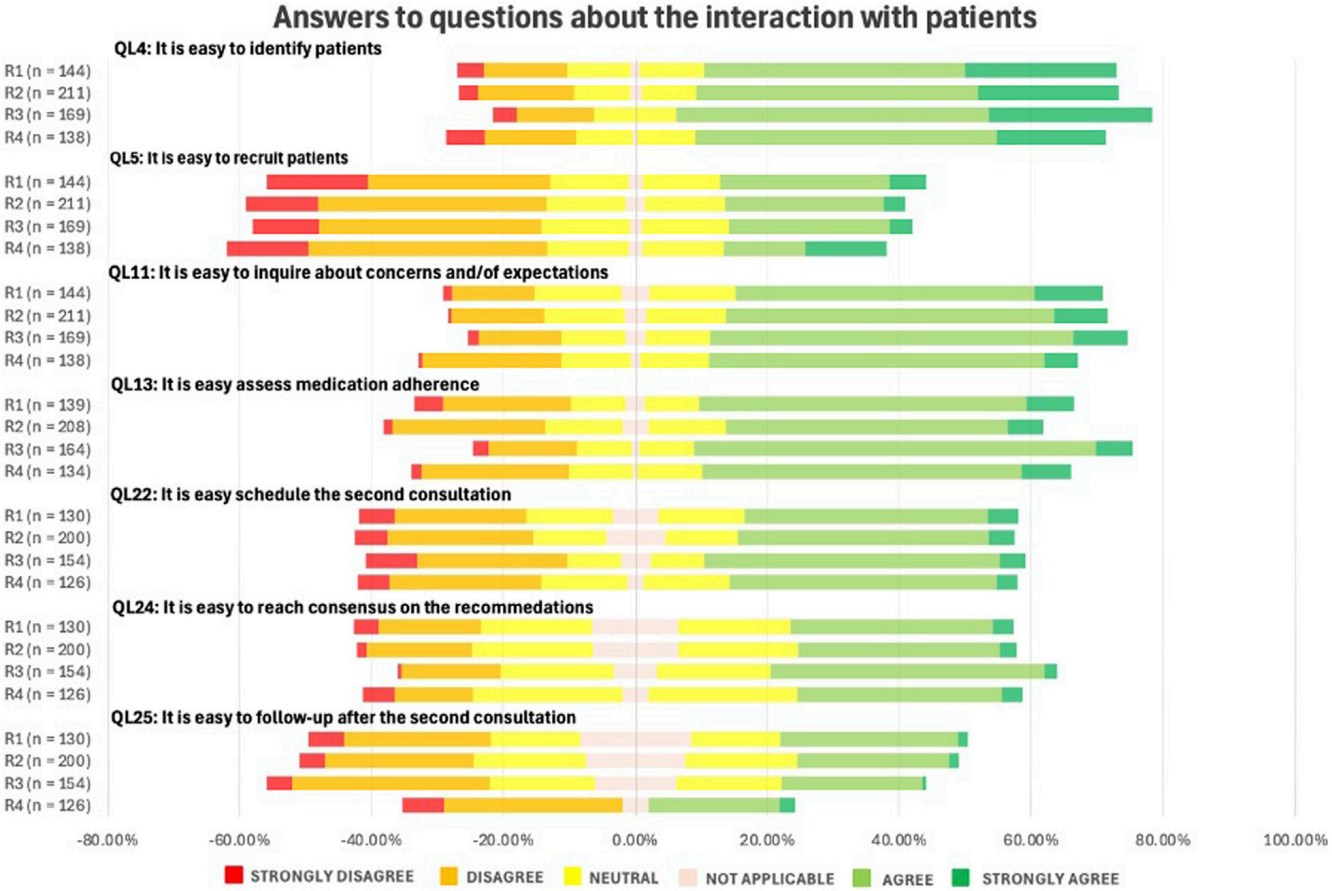
Likert Scale questions (QLs) about the pharmacists’ perceptions on the interaction with patients in the context of MR. (See in the appendix for full questionnaire).

As displayed in Figure 9, interaction with patients was generally perceived as relatively straightforward. Respondents agreed that identifying patients for MR2a (QL4) is easy, but reported greater difficulty in recruiting them for the service (QL5). Responses remained consistent across the different rounds (p-value = 0.978).

### 3.5 Qualitative evaluation

**FIGURE 10:**
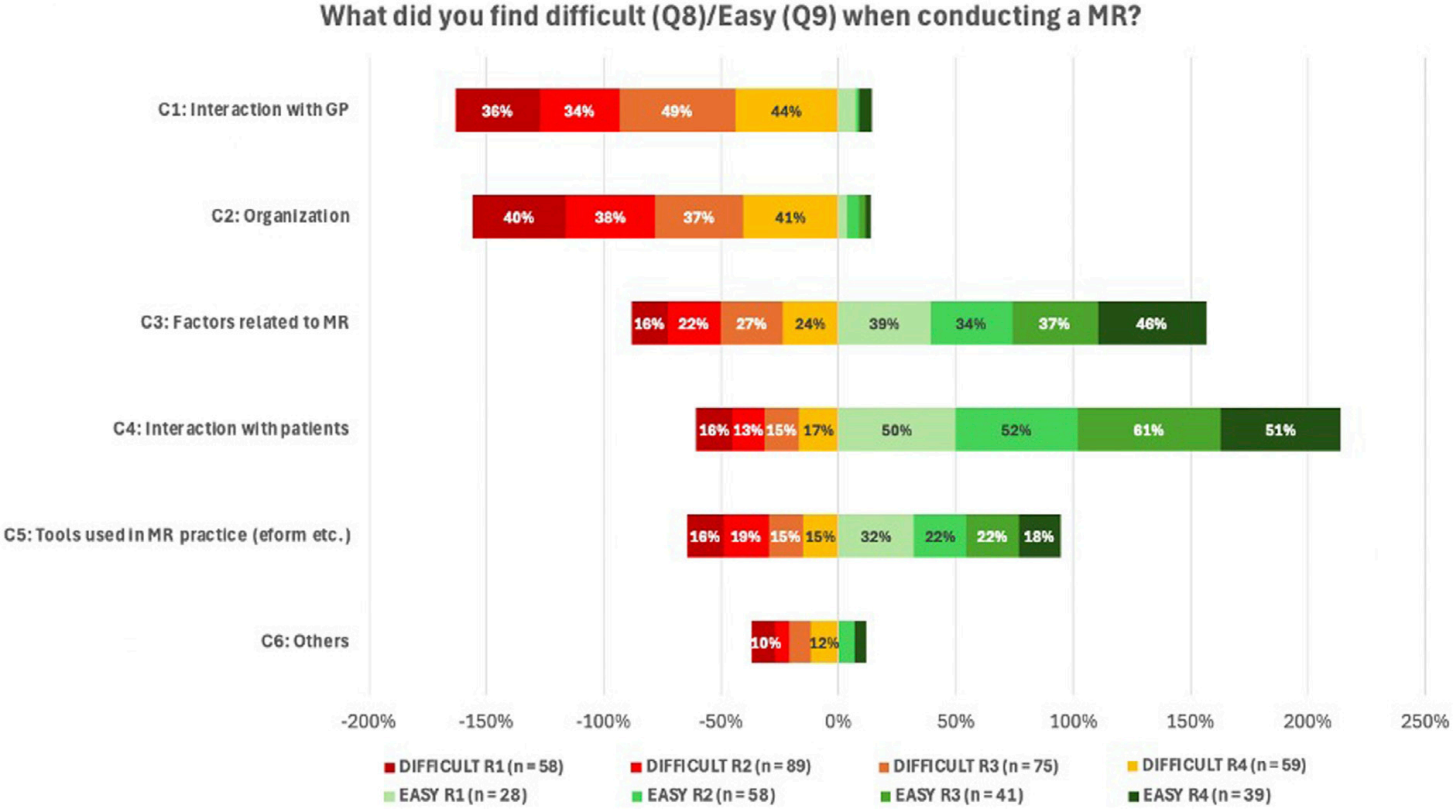
Overview of responses to Q8 and Q9 regarding challenges and facilitators in performing MR2a. Answers were categorized into six themes: C1 – Interaction with the GP; C2 – organizational aspects within the pharmacy (e.g., space, time, staff); C3 – the service itself (e.g., conducting the consultation, data analysis, drafting an action plan); C4 – collaboration with patients; C5 – tools used during MR2a (mainly the e-form, but also other resources); C6 – Others (e.g., lack of experience, legal framework). As these were open-ended questions, responses could fall into multiple categories, resulting in totals exceeding 100% per round.

Analysis of open-ended questions Q8 and Q9 (challenges and facilitators experienced when performing MR2a), as shown in Figure 10, confirms prior observations: Interactions with the GP and organization of the MR2a service were the most frequently cited challenges (response rates: 34%–49% and 37%–41%, respectively). In contrast, aspects directly related to MR2a (34%–46%) and interaction with the patients (50%–61%) were generally experienced as straightforward. Perceptions regarding the available tools for conducting MR2a, especially those linked to the e-form, were divided. Across the different rounds, 15%–19% of respondents perceived this aspect as difficult, while 18%–32% found it relatively easy.

To identify ways to support MR2a implementation, pharmacists indicated what additional support they required (Q10). As this question was not mandatory, response rates varied per round (R1: 37; R2: 67; R3: 54; R4: 42). Reported needs were grouped into five categories: legal framework, campaigns, in-pharmacy organization, collaboration with GPs and other factors (e.g., software, tools, e-form, training). An overview of these results is provided in the Supplementary Material.

## 4 Discussion

### 4.1 Participation rate and respondents’ characteristics

In round 1–4, respectively 210, 305, 226 and 213 participants consented to data analysis. The completion rates varied: among participants who completed more than 20% of the survey, response rates ranged from 76% to 85%, for those completing over 50%, rates ranged between 69% and 78% and full completion ranged from 51% to 70%, which is much higher than the typical 20%–30% for online surveys with over 15 questions (Liu and Wronski, 2017).

There were 4,714 pharmacies in Belgium in 2023 (SIRIUS_Insight, 2023). Approximately one in ten participated in this online survey, which is below the average online response rate of 44% (Wu et al., 2022). Nonetheless, the sample remains representative, as it includes a substantial number of pharmacists who had not yet conducted MR2A. Moreover, the participation of these specific group of pharmacists suggests an interest in MR2a service and its potential implementation. Furthermore, over half of participants exited the questionnaire at the language or consent stage, likely due to time constraints, as many pharmacists likely attempted to complete the survey during working hours.

### 4.2 Current adoption and future willingness to implement MR2a

#### 4.2.1 Pharmacists who have not yet implemented MR2a

Overall, 36%–49% of pharmacists had not yet implemented MR2a, aligning with international studies showing similarly low adoption rates (Nabergoj Makovec et al., 2018; Bradley et al., 2008; Niquille et al., 2010; Waltering et al., 2022; Abu Assab et al., 2022; Blenkinsopp et al., 2008; Brandt et al., 2020). For instance, a study performed in 2008 showed that nearly 75% of independent pharmacies in England had not implemented MRs after 2 years (Blenkinsopp et al., 2008; Brandt et al., 2020).

In this study, 15% of the participants who have not yet implemented MR2a clearly expressed reluctance, highlighting the need for better communication about its benefits (BCFI, 2023; RIZIV, 2024a). Pharmacy owner support is vital, studies show that engaged leadership boosts MR2a implementationt (Schulz et al., 2023; Abu Assab et al., 2022).

The main barrier to MR2a implementation were organizational difficulties, primarily due to staff shortages, single-pharmacist setting or high workloads. As in other healthcare settings, staff shortages often arise from financial constraints, recruitment difficulties, limited availability of qualified personnel and high workload demands (Hawthorne and Anderson, 2009). Structured planning, phased implementation and buddy systems have proven effective in other settings (Nabergoj Makovec et al., 2018). In R3 and R4 of this survey, over 100 pharmacists indicated a lack of experience and expressed their interest in receiving support through a buddy system, while 22 were willing to serve as buddies, highlighting its perceived value as a form of guidance. Furthermore, despite legal requirements in Belgium since 2009, the absence of a designated private area in the pharmacy remains a reported barrier (Federale_overheidsdienst_Justitie, 2009).

Time constraints pose a challenge for pharmacists managing MR2a alongside other responsibilities. The APB suggests scheduling fixed time slots, ideally during quieter periods or when additional staff is available (APB, 2024).

#### 4.2.2 Pharmacists that have performed MR2a

Most participants indicated performing at least one MR2a, with the most common range being one MR (13%–15%) or 2–5 MRs (18%–28%). Since reimbursement began in April 2023, over 500 pharmacists initiated MR2a, resulting in 11,530 reviews, according to APB (personal communication with IDW, email to author 7 July 2025), corresponding to uptake by 12% of the community pharmacists. This aligns with international benchmarks and reflecting a strong engagement from early adopters (APB, 2025b).

Although MR2a uptake appears to be slightly rising (personal communication with IDW, email to author 7 July 2025), variations in implementation volume may signal differences in engagement or potential quality differences. As this study did not assess MR2a quality, further analysis was not possible. Future work should integrate quality monitoring, such as report checklists and patient feedback, to safeguard clinical value and support sustainable reimbursement in Belgium (Robberechts et al., 2024; Robberechts et al., 2023a; Robberechts et al., 2023b; Robberechts et al., 2021; Tobback et al., 2025).

### 4.3 Education and training initiatives on medication review

Figures 3, 4 indicate that the median number of MR2a training sessions remained consistent (1,6–1,7) and that training facilitates higher MR2a uptake. However, some trained pharmacists still do not perform MR2a due to fear and organizational barriers, while others conduct MR2a without training, raising concerns about quality. Recent graduates may partly explain this, but enforcement of mandatory training for inexperienced pharmacists remains inconsistent (RIZIV, 2024a).

### 4.4 Data about the in-practice implementation

#### 4.4.1 The duration of the patient consultation

The most commonly reported duration of patient consultations ranged between 15 and 60 min, with complex cases exceeding 60 min, consistent with SIMENON project findings (Wuyts, 2019). However, cases exceeding 60 min raise financial feasibility concerns, as these complex cases benefit most from MR2a. A Polish study similarly reported average consultation times of 38 min for simple cases, increasing to 71 min in complex cases (Merks et al., 2022). To enhance efficiency, APB recommends informing patients about the 30-min consultation and employing concise communication strategies (Robinson, 2022). Consultations under 15 min may compromise quality, especially given the €10117 reimbursement per MR2a by the National Institute for Health and Disability Insurance (RIZIV/INAMI) (RIZIV, 2024a; RIZIV, 2024b).

#### 4.4.2 Information-sources and tools

Pharmacists routinely consult standard pharmacotherapeutic sources, including national databases (e.g., BCFI (BCFI, 2024), Phil, (APB, 2023), the SmPC, (EMA, 2025), and clinical tools embedded in the e-form (APB, 2025c)). For older adults, tools such as GheOP3S and STOPP/START criteria, are commonly applied (Foubert et al., 2021; BCFI, 2023; Ghent, 2023; O'Mahony et al., 2023; Gallagher et al., 2008; Hill-Taylor et al., 2013; Barry et al., 2007). Subscription-based resources, like UpToDate^®^, are less frequently used (Kluwer).

Over 70% of pharmacists consult four or more S/Ts during MR2a, with only 0%–5% proceed without any, potentially compromising quality. Multiple sources are essential for evidence-based analysis. These findings highlight the need for a systematic quality assessment (Robberechts, 2024; Robberechts et al., 2023a) and suggest that governmental support, through funding or licensing, could promote broader source use and MR2a quality.

#### 4.4.3 Collaboration with GPs

Communication with GPs remains a key challenge in MR2a implementation, with no significant change over time (p = 0.989), indicating limited progress. Consistent with international evidence, collaboration barriers persist as many GPs are unfamiliar with MR2a, highlighting the importance of effective communication to address key concerns and optimizing MR2a (Nabergoj Makovec et al., 2018; Bradley et al., 2008; Niquille et al., 2010; Abu Assab et al., 2022; Waltering et al., 2022; Latif, 2017; Eickhoff et al., 2021; APB, 2025c).

Secure, user-friendly communication platforms are urgently needed, as existing platforms (e.g., eHealthbox) are poorly integrated and non-GDPR-compliant alternatives (e.g., email, WhatsApp) cannot be used (eGezondheid; KOVAG, 2024). In Belgium, Medical-Pharmaceutical Concertation (MPC) was introduced to facilitate communication between GPs and community pharmacists (Damiaens et al., 2021). To support this initiative, APB developed frameworks such as MPC *MR2a and communication in Primary Care* to streamline information exchange, clarify roles and improve overall efficiency (APB, 2025c).

#### 4.4.4 Interaction with patients

Patient interaction was generally reported as straightforward, but recruiting patients and follow-up remain challenging. MR2a is still relatively unknown to the public, hampering recruitment (Waltering et al., 2022). Effective follow-up is crucial to prevent new DRPs and assess outcomes (Montgomery et al., 2008; MacCallum and Dolovich, 2018). In many cases, a quick check-in during a subsequent pharmacy visit may suffice as follow-up. Overall, the persistent challenges suggest enduring barriers and possible saturation among early adopters.

### 4.5 Qualitative evaluations

Pharmacists frequently reported barriers related to GP collaboration (36%–49%) and organizational issues, such as time constraints and staffing (37%–41%), largely due to limited GP availability and lack of compensation, aligning with international data (Gallagher and Gallagher, 2012; Zielinska-Tomczak et al., 2021).

Factors related to conducting a MR2a, such as the e-form, software and pharmacotherapeutic analysis were perceived both challenging and feasible. Following user feedback, APB revised the e-form in September 2024 (FarmaFlux, 2024). Future evaluations should assess the impact of these changes and the need for further improvements.

In contrast, patient interaction was generally perceived positively, though recruitment and managing consultation time remain difficult. Yet, studies have shown that pharmacists generally communicate well with patients (Kerr et al., 2021). Traditionally focused on transferring medication information, their role has evolved towards patient-centered care, making strong communication skills essential (Jacob et al., 2019). Simulated patient training could help pharmacists overcome hesitancy and improve patient communication in MR2a practice (Kerr et al., 2021).

Pharmacists identified the need for additional support in MR2a implementation, including administrative simplification, expanded training, centralized resources and secure communication with the GP. Successful deployment of MR initiatives necessitates structured guidance and endorsement from governing bodies; in its absence, uptake remains limited, especially as many pharmacists are reluctant to begin offering the service. Much of the requested support already exists but remains underutilized, likely due to limited awareness among pharmacists. There are various training programs available, ranging from basic education to advanced masterclasses (SSPF, 2025; IPSA, 2025). Some pharmacists requested a higher reimbursement to keep this service viable, though evidence suggests financial incentives a line are insufficient (Waltering et al., 2022; Robberechts et al., 2021).

Lastly, national and/or regional campaigns were requested to raise awareness of MR2a among GPs and the general public. As part of national health-insurance agreements, Belgium has a single payer system (Lynch and Vermorken, 2021), pharmacists are obliged to offer the service if patients meet the requirements. APB provides promotional materials on their website (APB, 2025c). Additionally, there is a need for in-pharmacy support, including mentorship visits from experienced MR2a colleagues and peer-exchange platforms. In response, some professional associations have initiated coaching projects, where pharmacists with MR2a expertise support colleagues in adopting and optimizing the service (personal communication with Verrue C).

### 4.6 Study limitations and strengths

This study has several limitations. Although responses with >50% completion were included, fully completed questionnaires yielded very similar results, supporting validity of including partial responses. Limiting the overall length of the survey constrained our ability to research these additional dimensions and further reduction in survey length might have improved response and completion rates This study could not assess the quality of the MR2a interventions, patient selection or other specific MR2a characteristics. Future research should address these aspects and explore tools to evaluate service quality. In addition, only limited demographic information was obtained from participants, which restricted the analysis of how MR2a-related experiences might, for example, differ according to pharmacists’ roles in community pharmacies.

Despite these limitations, this study offers several strengths. Its longitudinal four-rounded design enabled observation and analysis of implementation trends over time. The rigoursly developed bilingual survey, created in collaboration with Belgian universities and professional organizations, ensured relevance and clarity. The study’s mixed-methods approach and inclusive sampling enhanced representativeness and reliability, providing context-sensitive insights into MR2a integration in Belgian pharmacy practice, with implications for international medication review initiatives.

## 5 Conclusion

Participant’s opinions on GP collaboration and interaction with patients remained consistent across rounds, suggesting that key facilitators and barriers are persistent over time. Progress in the implementation of MR2a in Belgian community pharmacies remains limited. Major barriers include time constraints, staff shortages, insufficient collaboration with GPs and lack of practical support tools on communication. However, targeted training initiatives and positive patient interactions act as important facilitators, which motivate pharmacists to engage more actively in MR2a.

Continued support by different stakeholders, professional associations and educational institutions is essential. Implementing periodic surveys of pharmacists are a feasible and valuable method to track implementation progress and inform adjustments to newly introduced services.

## Data Availability

The datasets presented in this study can be found in online repositories. The names of the repository/repositories and accession number(s) can be found in the article/Supplementary Material.
