# Supplementary material for: Implementation of medication review type 2a in community pharmacies: a longitudinal survey study
Source: Front Pharmacol. 2025 Nov 12;16:1685922. doi: 10.3389/fphar.2025.1685922 (PMC12648213; doi:10.3389/fphar.2025.1685922)

# Appendix

## Online questionnaire in Qualtrics (English)

**Q0. Consent of the participant**

I declare that I have been informed in a clear and comprehensible manner about the nature, method and purpose of this survey. I understand that all data will be treated with strict confidentiality.

- I give my consent
- I do not give my consent

*Skip To: End of Survey If Q0. = I do not give my consent*

**Part 1: General Information**

Please answer the following questions.

**Q1. When was the last time you supervised a pharmacist intern?**

- Never
- 5 or more years ago
- Less than 5 years ago

**Q1e.** **What is the postal code of the pharmacy where you work? (optional to answer)**

**Q2. When did you graduate**

- 5 or more than 5 years ago
- Less than five years ago

**Q3. What education did you pursue to prepare for conducting medication reviews?**

- None
- IPSA Medicatienazicht Basic IPSA (2019 – 2024)
- IPSA Advanced Medication Review
- Opleidingsreeks Meduplace – Medication Review Type 3
- Webinarreeks IPSA/APB – Medicatienazicht – 2023
- Apinto-avond Medicatienazicht Workshop (IPSA)
- MFO medicatienazicht type 2a en communicatie in eerste lijn ...
- MFO medication review type III – de weg naar veilig en rationeel ...
- IPSA Webinar GGG Medicatienazicht – Charlotte Verrue (april ...
- SSPF Bilan de médication Basic (2019 – 2022)
- Webinaires SSPF Revue de la médication 2023 (protocole – ...
- Best-Seller des outils de revue de médication – Adéquation des ...
- Atelier SSPF - Revue de la médication en pratique – formation ...
- Week-end Masterclass – Revue de la médication: de la théorie à ...
- CMP - Gestion interdisciplinaire de la polymédication chez le ...
- Atelier pratique en soirée: Revue de la médication – kit de ...
- E-learning: Revue de la médication : de la théorie à la pratique ...
- Ateliers: Revue de la médication en pratique – en ligne (2023)
- Others:

**Q4. How many medication reviews (in general) have you already conducted, aside from reimbursement?**

- None
- 1 Medication review
- 2-5 Medication reviews
- 6-10 Medication review
- 11-20 Medication reviews
- > 20 Medication reviews, specify:

*Skip To: Q4b If Q4 = None*

**Q5. How many individual GPs do you collaborate with on medication reviews?**

- None
- 1 GP
- 2 GPs
- 3 GPs
- > 3 GPs

*Display this question:*

*If Q4 = None*

**Q4b. What is the main reason you have not yet started conducting medication reviews?**

- I want to start and have the necessary competencies, but I can’t get it organized.
- I want to start, but I have cold feet.
- I want to start, but there are other reasons, please specify:
- I do not wish to conduct medication reviews.

*Skip To: Q50 If Q4b = I do not wish to conduct medication reviews*

*Display this question:*

*If Q4b = I want to start and have the necessary competencies, but I can’t get it organized*

**Q4c. What prevents the organization of conducting medication reviews in your pharmacy?**

- I work alone in the pharmacy.
- I do not have sufficient staff.
- I do not have enough time for this.
- My pharmacy does not have a confidential consultation area
- My software does not provide access to the e-form.
- There are other reasons, please specify:

*Display this question:*

*If Q4b = I do not wish to conduct medication reviews*

**Q50.: Why have you decided not to conduct medication reviews yet? (Answering is optional, but all responses are of course very valuable)**

**Q51.: What would help you to get started? (Multiple options possible)**

- I would start if clear agreements were in place with GPs.
- I would start if there were better financial compensation.
- I would start if I had more staff.
- I would start if I could complete the first cases together with a mentor/buddy.
- I would start if organizational support were available.
- I would start if … (other – please specify):
- For now, I stick to my current position.

**Part 2: How was your experience in going through the different steps of a medication review?**

Please answer based on the **average situation**, as it typically occurs, rather than focusing on exceptional cases.

If you have **not** yet conducted a medication review, **please indicate to what extent you agree with the statement.**

**QL1.: It is difficult to free up time and staff to conduct medication reviews.**

- Strongly agree
- Agree
- Neutral
- Disagree
- Strongly disagree
- Not Applicable

**QL2.: It is difficult to invite patients for a medication review.**

- Strongly agree
- Agree
- Neutral
- Disagree
- Strongly disagree
- Not Applicable

**QL3.: It is easy to inform the local GP about medication review.**

- Strongly agree
- Agree
- Neutral
- Disagree
- Strongly disagree
- Not Applicable

**QL4.: It is easy to identify patients who are eligible for a medication review.**

- Strongly agree
- Agree
- Neutral
- Disagree
- Strongly disagree
- Not Applicable

**QL5.: It is easy to recruit patients* to conduct medication reviews.**

*This refers to patients who meet the criteria for a medication review and agree to participate.

- Strongly agree
- Agree
- Neutral
- Disagree
- Strongly disagree
- Not Applicable

**QL6.: It is easy to list the relevant non-prescription medicines and health products.**

- Strongly agree
- Agree
- Neutral
- Disagree
- Strongly disagree
- Not Applicable

**QL7.: It is easy to inform the GP before the patient interview that you will conduct a medication review.**

- Strongly agree
- Agree
- Neutral
- Disagree
- Strongly disagree
- Not Applicable

**QL8.: It is easy to collect information* form the treating GP prior to the patient interview.**

*This includes lab values (e.g. renal function) and indications not deducible from the medication history.

- Strongly agree
- Agree
- Neutral
- Disagree
- Strongly disagree
- Not Applicable

**QL9.: It is difficult to determine the conditions for which the patient uses their medications (reason for use).**

- Strongly agree
- Agree
- Neutral
- Disagree
- Strongly disagree
- Not Applicable

**QL10.: It is difficult to explain the purpose and added value of the medication review to the patient.**

- Strongly agree
- Agree
- Neutral
- Disagree
- Strongly disagree
- Not Applicable

**QL11.: It is easy to ask the patient about their concerns and/or expectations regarding their medication.**

- Strongly agree
- Agree
- Neutral
- Disagree
- Strongly disagree
- Not Applicable

**QL12.: It is difficult to ask the patient about adverse effects of their medication.**

- Strongly agree
- Agree
- Neutral
- Disagree
- Strongly disagree
- Not Applicable

**QL13.: It is easy to ask the patient about their adherence.**

- Strongly agree
- Agree
- Neutral
- Disagree
- Strongly disagree
- Not Applicable

**QL14.: It is easy to detect interactions.**

- Strongly agree
- Agree
- Neutral
- Disagree
- Strongly disagree
- Not Applicable

**QL15.: It is difficult to assess whether the problems detected by GheOP3S in the e-form are relevant to the patient.**

- Strongly agree
- Agree
- Neutral
- Disagree
- Strongly disagree
- Not Applicable

**QL16.: It is difficult to select which problems should be addressed first.**

- Strongly agree
- Agree
- Neutral
- Disagree
- Strongly disagree
- Not Applicable

**QL17.: It is easy to formulate recommendations to the GP for identified problems.**

- Strongly agree
- Agree
- Neutral
- Disagree
- Strongly disagree
- Not Applicable

**QL18.: It is easy to formulate recommendations to the patient when preparing the action plan for identified problems.**

- Strongly agree
- Agree
- Neutral
- Disagree
- Strongly disagree
- Not Applicable

**QL19.: It is easy to make the pharmaceutical report available to the GP.**

- Strongly agree
- Agree
- Neutral
- Disagree
- Strongly disagree
- Not Applicable

**QL20.: It is easy to obtain feedback from the GP on the pharmaceutical report.**

- Strongly agree
- Agree
- Neutral
- Disagree
- Strongly disagree
- Not Applicable

**QL21.: It is easy to discuss the contents of the pharmaceutical report with the GP.**

- Strongly agree
- Agree
- Neutral
- Disagree
- Strongly disagree
- Not Applicable

**QL22.: It is easy to schedule a suitable moment with the patient to discuss the results.**

- Strongly agree
- Agree
- Neutral
- Disagree
- Strongly disagree
- Not Applicable

**QL23.: It is difficult to discuss the results of the medication review with the patient (second interview).**

- Strongly agree
- Agree
- Neutral
- Disagree
- Strongly disagree
- Not Applicable

**QL24.: It is easy to reach consensus with the patient on the proposed recommendations.**

- Strongly agree
- Agree
- Neutral
- Disagree
- Strongly disagree
- Not Applicable

**QL25.: It is easy to follow-up with the patient after the second consultation.**

- Strongly agree
- Agree
- Neutral
- Disagree
- Strongly disagree
- Not Applicable

**Part 3: Other questions about the medication review process**

**Q6.: What tools or resources do you regularly use during the medication review?**

- GheOP3S
- STOPP/START-criteria
- PhiL (PhiL.apb.be)
- Belgian Center for Pharmacotherapeutic Information (BCFI)
- Summary of Product Characteristics (SmPC) (= scientific package leaflet)
- Guidelines (e.g. Domus Medica, NHG-richtlijnen,SSMG ...)
- Pharmacotherapeutic compass (NL) (www.farmacotherapeutischkompas.nl)
- Prescrire (www.prescrire.org)
- UpToDate® (www.uptodate.com)
- Medicines Complete® (www.medicinescomplete.com)
- Vidal® (www.vidal.fr)
- Others: please specify:
- Not Applicable

**Q7.: How long did the patient consultation typically last on average?**

- < 15 minutes
- 15 to 30 minutes
- 31 to 45 minutes
- 46 to 60 minutes
- 61 to 75 minutes
- 75 minutes
- Not applicable

**Q8.: What did you find difficult when conducting a medication review? (answering is optional)**

**Q9.: What did you find easy when conducting a medication review? (answering is optional)**

**Q10.: What (additional) support or tools would you like to have to conduct medication reviews? (answering is optional)**

**Q11.: If applicable, University of the intern pharmacist for the current academic year.**

- **Not applicable (no intern this academic year)**
- **KU Leuven**
- **UAntwerp**
- **UCLouvain**
- **ULB**
- **ULiège**
- **UMons**
- **University of Ghent**
- **Vrije Universiteit Brussel**

**Qe2.: The last 2 questions (multiple choice and open-ended) are about the implementation of a buddy system, where a colleague pharmacist provides assistance in conducting medication reviews.**

- I do not have sufficient experience yet, but I do not wish to receive support form a buddy.
- I do not have sufficient experience yet and would like the support from a buddy
- I have sufficient experience with medication reviews, but I would not volunteer as a buddy.
- I have sufficient experience with medication reviews and I would like to volunteer as a buddy.
- Others:

**Qe3.: How do you envision the buddy system being practically organized? (Answering is optional)**

**Q12: Pseudo-identification of the responsible pharmacist.**

Please create a unique code for yourself so that your responses can be anonymously linked across future survey rounds (without allowing researchers to identify you). The code consists of the first two letters of your mother’s first name, the first two letters of your mother’s last name and the last digit of your birth year. For example: MAGA9.

## Online vragenlijst in Qualtrics

**Q.0 Toestemming van de vrijwilliger**

Ik verklaar op een voor mij duidelijke wijze te zijn ingelicht over de aard, methode en doel van deze enquête.

Ik weet dat de gegevens en resultaten strikt vertrouwelijk zullen worden verwerkt.

- Ik geef toestemming
- Ik geef geen toestemming

*Skip To: End of Survey If Q.0 = Ik geef geen toestemming*

**Deel 1: Algemene gegevens:**

Gelieve de volgende vragen te beantwoorden.

**Q1. Wanneer heb je het laatst een apotheker-stagiair begeleid?**

- Nooit
- 5 of meer dan 5 jaar geleden
- Minder dan 5 jaar geleden

**Q1e. Wat is de Postcode van de apotheek waarin u werk (antwoorden optioneel)?**

**Q2. Wanneer ben je afgestudeerd?**

- 5 of meer dan 5 jaar geleden
- Minder dan 5 jaar geleden

**Q3 Welke opleiding heb je gevolgd om je voor te bereiden op het uitvoeren van medicatienazichten?**

- Geen
- IPSA Medicatienazicht Basic IPSA (2019 – 2024)
- IPSA Advanced Medication Review
- Opleidingsreeks Meduplace – Medication Review Type 3
- Webinarreeks IPSA/APB – Medicatienazicht – 2023
- Apinto-avond Medicatienazicht Workshop (IPSA)
- MFO medicatienazicht type 2a en communicatie in eerste lijn ...
- MFO medication review type III – de weg naar veilig en rationeel ...
- IPSA Webinar GGG Medicatienazicht – Charlotte Verrue (april ...
- SSPF Bilan de médication Basic (2019 – 2022)
- Webinaires SSPF Revue de la médication 2023 (protocole – ...
- Best-Seller des outils de revue de médication – Adéquation des ...
- Atelier SSPF - Revue de la médication en pratique – formation ...
- Week-end Masterclass – Revue de la médication: de la théorie à ...
- CMP - Gestion interdisciplinaire de la polymédication chez le ...
- Atelier pratique en soirée: Revue de la médication – kit de ...
- E-learning: Revue de la médication : de la théorie à la pratique ...
- Ateliers: Revue de la médication en pratique – en ligne (2023)
- Andere:

**Q4. Hoeveel medicatienazichten (in het algemeen) heb je reeds uitgevoerd, los van de terugbetaling? Dit houdt ook Medication reviews in die plaatsvonden vóór het in voege gaan van de terugbetaling in april 2023, waarbij minstens een patiëntengesprek en een farmacotherapeutische analyse werd uitgevoerd.**

- Geen
- 1 medicatienazicht
- 2 tot 5 medicatienazichten
- 6 tot 10 medicatienazichten
- 11 tot 20 medicatienazichten
- >20, specifieer:

*Skip To: Q4b If Q4 = Geen*

**Q5**. **Met hoeveel individuele artsen werk je samen rond medicatienazichten?**

- Geen artsen
- 1 arts
- 2 artsen
- 3 artsen
- > 3 artsen

*Display this question:*

*If Q4 = Geen*

**Q4b Wat is de belangrijkste reden waarom je nog niet bent gestart met het uitvoeren van medicatienazichten?**

- Ik wil starten en beschik over de nodige competenties, maar ik krijg dit niet georganiseerd
- Ik wil starten, maar ik heb koudwatervrees
- Ik wil starten, maar er zijn een andere redenen, specifieer:
- Ik wens geen medicatienazichten uit te voeren

*Skip To: Q50 If Q4b = ik wens geen medicatienazichten uit te voeren*

**Q4c Wat verhindert in Uw apotheek de organisatie van het uitvoeren van medicatienazichten?**

- Ik sta alleen in de apotheek
- Ik beschik over onvoldoende personeel
- Ik heb onvoldoende tijd hiervoor
- Mijn apotheek beschikt niet over een vertrouwelijkheidshoekje
- Ik heb geen toegang via mijn softwarepakket tot de eform
- Er zijn andere redenen, specifieer:

*Display this question:*

*If Q4b = ik wens geen medicatienazichten uit te voeren*

**Q50 Waarom heeft u besloten om nog geen medicatienazichten uit te voeren? (antwoorden is optioneel, maar alle antwoorden zijn natuurlijk heel waardevol!)**

**Q51 Wat zou u helpen om toch te starten? (meerdere opties mogelijk)**

- Ik zou starten indien er duidelijke afspraken zijn met artsen
- Ik zou starten indien er een betere vergoeding was
- Ik zou starten indien ik meer personeel had
- Ik zou starten indien ik de eerste casussen samen met een buddy kan uitvoeren
- Ik zou starten indien er op organisatorisch vlak begeleiding was
- Ik zou starten indien, andere......
- Ik blijf voorlopig bij mijn standpunt

**Deel 2: Hoe was je ervaring bij het doorlopen van de verschillende stappen van een medicatienazicht?**

Denk bij het antwoorden aan de **gemiddelde situatie**, dus zoals die zich meestal voordoet, en niet te veel aan één uitzonderlijke situatie.

Indien je nog geen medicatienazicht hebt uitgevoerd, **gelieve aan te duiden in hoeverre je akkoord gaat met de stelling.**

**QL1 Het is moeilijk om tijd en personeel vrij te maken om medicatienazichten uit te voeren**

- helemaal akkoord
- akkoord
- neutraal
- niet akkoord
- helemaal niet akkoord
- niet van toepassing

**QL2 Het is moeilijk om patiënten uit te nodigen voor een medicatienazicht**

- helemaal akkoord
- akkoord
- neutraal
- niet akkoord
- helemaal niet akkoord
- niet van toepassing

**QL3 Het is eenvoudig om de artsen uit de omgeving te informeren over het medicatienazicht**

- helemaal akkoord
- akkoord
- neutraal
- niet akkoord
- helemaal niet akkoord
- niet van toepassing

**QL4 Het is eenvoudig om de patiënten te identificeren die in aanmerking komen voor een medicatienazicht**

- helemaal akkoord
- akkoord
- neutraal
- niet akkoord
- helemaal niet akkoord
- niet van toepassing

**QL5 Het is gemakkelijk om patiënten te rekruteren* om medicatienazichten uit te voeren**

*Dit gaat over patiënten die aan de voorwaarden voldoen om in aanmerkingen te komen voor een medicatienazicht en die ook instemmen om hier aan deel te nemen.

- helemaal akkoord
- akkoord
- neutraal
- niet akkoord
- helemaal niet akkoord
- niet van toepassing

**QL6 Het is eenvoudig om de relevante niet voorgeschreven geneesmiddelen en gezondheidsproducten op te lijsten**

- helemaal akkoord
- akkoord
- neutraal
- niet akkoord
- helemaal niet akkoord
- niet van toepassing

**QL7 Het is eenvoudig om de arts te informeren vóór het patiënten gesprek dat je een medicatienazicht bij zijn patiënt zal uitvoeren**

- helemaal akkoord
- akkoord
- neutraal
- niet akkoord
- helemaal niet akkoord
- niet van toepassing

**QL8 Het is eenvoudig om van de behandelende arts informatie* te verzamelen vóór het patiëntengesprek**

*Met informatie worden labowaarden (bv. nierfunctie) en indicaties, die niet uit de medicatiehistoriek kan worden afgeleid, bedoeld.

- helemaal akkoord
- akkoord
- neutraal
- niet akkoord
- helemaal niet akkoord
- niet van toepassing

**QL9 Het is moeilijk te achterhalen voor welke aandoeningen de patiënt zijn geneesmiddelen gebruikt (reason for use)**

- helemaal akkoord
- akkoord
- neutraal
- niet akkoord
- helemaal niet akkoord
- niet van toepassing

**QL10 Het is moeilijk om het doel en de meerwaarde van het medicatienazicht aan de patiënt uit te leggen**

- helemaal akkoord
- akkoord
- neutraal
- niet akkoord
- helemaal niet akkoord
- niet van toepassing

**QL11 Het is eenvoudig om de bezorgdheden en/of verwachtingen van de patiënt ten aanzien van zijn geneesmiddelen te bevragen**

- helemaal akkoord
- akkoord
- neutraal
- niet akkoord
- helemaal niet akkoord
- niet van toepassing

**QL12 Het is moeilijk om de patiënt te bevragen naar de ongewenste effecten van zijn medicatie**

- helemaal akkoord
- akkoord
- neutraal
- niet akkoord
- helemaal niet akkoord
- niet van toepassing

**Q13 Het is gemakkelijk om de therapietrouw van de patiënt te bevragen**

- helemaal akkoord
- akkoord
- neutraal
- niet akkoord
- helemaal niet akkoord
- niet van toepassing

**QL14 Het is gemakkelijk om interacties op te sporen**

- helemaal akkoord
- akkoord
- neutraal
- niet akkoord
- helemaal niet akkoord
- niet van toepassing

**QL15 Het is moeilijk om te beoordelen of de problemen die door GheOP3S in het eform worden gedetecteerd, relevant zijn voor de patiënt.**

- helemaal akkoord
- akkoord
- neutraal
- niet akkoord
- helemaal niet akkoord
- niet van toepassing

**QL16 Het is moeilijk om de problemen die eerst moeten aangepakt worden te selecteren**

- helemaal akkoord
- akkoord
- neutraal
- niet akkoord
- helemaal niet akkoord
- niet van toepassing

**QL17 Het is makkelijk om aanbevelingen naar de arts te formuleren voor vastgestelde problemen**

- helemaal akkoord
- akkoord
- neutraal
- niet akkoord
- helemaal niet akkoord
- niet van toepassing

**QL18 Het is makkelijk om bij de opmaak van het actieplan om aanbevelingen naar de patiënt te formuleren voor vastgestelde problemen**

- helemaal akkoord
- akkoord
- neutraal
- niet akkoord
- helemaal niet akkoord
- niet van toepassing

**QL19 Het is eenvoudig om het farmaceutisch rapport ter beschikking te stellen aan de arts**

- helemaal akkoord
- akkoord
- neutraal
- niet akkoord
- helemaal niet akkoord
- niet van toepassing

**QL20 Het is eenvoudig om een reactie van de arts te bekomen op het farmaceutisch rapport**

- helemaal akkoord
- akkoord
- neutraal
- niet akkoord
- helemaal niet akkoord
- niet van toepassing

**QL21 Het is eenvoudig om over de inhoud van het farmaceutisch rapport te overleggen met de arts**

- helemaal akkoord
- akkoord
- neutraal
- niet akkoord
- helemaal niet akkoord
- niet van toepassing

**QL22 Het is eenvoudig om met de patiënt een geschikt moment te plannen om de resultaten te bespreken**

- helemaal akkoord
- akkoord
- neutraal
- niet akkoord
- helemaal niet akkoord
- niet van toepassing

**QL23 Het is moeilijk om met de patiënt de resultaten van het medicatienazicht te bespreken (gesprek 2)**

- helemaal akkoord
- akkoord
- neutraal
- niet akkoord
- helemaal niet akkoord
- niet van toepassing

**QL24 Het is eenvoudig om met de patiënt een consensus te bekomen over de voorgestelde aanbevelingen**

- helemaal akkoord
- akkoord
- neutraal
- niet akkoord
- helemaal niet akkoord
- niet van toepassing

**QL25 Het is gemakkelijk om de verdere opvolging van de patiënt te doen na het tweede gesprek**

- helemaal akkoord
- akkoord
- neutraal
- niet akkoord
- helemaal niet akkoord
- niet van toepassing

**Deel 3: Ander vragen over het medicatie-nazicht process**

**Q6. Welke tools of bronnen gebruik je regelmatig tijdens het medicatienazicht?**

- GheOP3S
- STOPP/START-criteria
- PhiL (PhiL.apb.be)
- Het Belgisch Centrum voor Farmacotherapeutische Informatie (BCFI)
- Samenvatting van de kenmerken van het product (SKP) (= wetenschappelijke bijsluiter)
- Behandelrichtlijnen (bv Domus Medica, NHG-richtlijnen,SSMG …)
- Farmacotherapeutisch Kompas (NL) (www.farmacotherapeutischkompas.nl)
- Prescrire (www.prescrire.org)
- UpToDate® (www.uptodate.com)
- Medicines Complete® (www.medicinescomplete.com)
- Vidal® (www.vidal.fr)
- Andere: specifieer
- niet van toepassing

**Q7. Hoelang duurde het patiëntengesprek gemiddeld?**

- < 15 minuten
- 15 tot 30 minuten
- 31 tot 45 minuten
- 46 tot 60 minuten
- 61 tot 75 minuten
- > 75 minuten
- niet van toepassing

**Q8 Wat vond je moeilijk bij het uitvoeren van een medicatienazicht? (antwoorden niet verplicht)**

**Q9 Wat vond je makkelijk bij het uitvoeren van een medicatienazicht? (antwoorden niet verplicht)**

**Q10 Welke (bijkomende) ondersteuning of hulpmiddelen zou je wensen om medicatienazichten uit te voeren? (antwoorden niet verplicht)**

**Q11 Indien van toepassing: Universiteit van de stagiair-apotheker van het lopende academiejaar**

- Niet van toepassing (geen stagair dit academiejaar)
- KU Leuven
- UAntwerpen
- UCLouvain
- ULB
- ULiège
- UMons
- Univerisiteit Gent
- Vrije Universiteit Brussel

**Qe2 De 2 laatste vragen (MCQ en open vraag) gaan over de implementering van een buddy- systeem waarbij een collega-apotheker hulp biedt bij het uitvoeren van medicatienazichten.**

- Ik heb nog onvoldoende ervaring, maar ik wens geen steun van een buddy
- Ik heb nog onvoldoende ervaring en zou graag de steun willen van een buddy
- Ik heb genoeg ervaring met medicatienazichten, maar ik zou mij niet opgeven als een buddy
- Ik heb genoeg ervaring met medicatienazichten en zou graag andere apothekers willen steunen als een buddy
- Andere: …

**Qe3 Hoe ziet u het systeem van een buddy praktisch georganiseerd? (antwoorden optioneel)**

**Q12 Pseudo-identificatie van de verantwoordelijke apotheker:**

Maak een unieke code voor jezelf aan zodat je antwoorden op vragenlijsten in toekomstige enquêterondes anoniem kunnen worden gekoppeld. (Dus zonder dat de onderzoekers je identiteit kunnen achterhalen.) De code bestaat uit de eerste twee letters van de voornaam en achternaam van je moeder, gevolgd door het laatste cijfer van jouw geboortejaar. Bijvoorbeeld: MAGA9

## Supplementary Data: Tables and Figures

**Extra data on language preference (section 3.1)**

**Table 1 Language preferences across rounds.**

| **Language preference** | | |
| --- | --- | --- |
|  | **French** | **Dutch** |
| **R1 (n = 150)** | 47% | 53% |
| **R2 (n = 237)** | 47% | 53% |
| **R3 (n = 174)** | 37% | 63% |
| **R4 (n = 147)** | 31% | 69% |

**Extra data on reasons why participants did not yet perform MRs (section 3.2)**

**Table 2 Overview of responses to question Q4b: What is the main reason you have not yet started conducting medication reviews?**

|  | **I do not wish to perform MRs** | **I can’t get it organized** | **I have cold feet** | **Others** |
| --- | --- | --- | --- | --- |
| R1(n = 60) | 10% | 37% | 8% | 45% |
| R2 (n = 115) | 23% | 21% | 19% | 37% |
| R3 (n = 72) | 7% | 21% | 17% | 56% |
| R4 (n = 53) | 17% | 28% | 17% | 38% |

**Extra data on the additional support pharmacists would like to receive (section 3.5)**

**Figure 1: Answers to the open-ended question Q10: What (additional) support or tools would you like to have to conduct medication reviews? (answering is optional)**


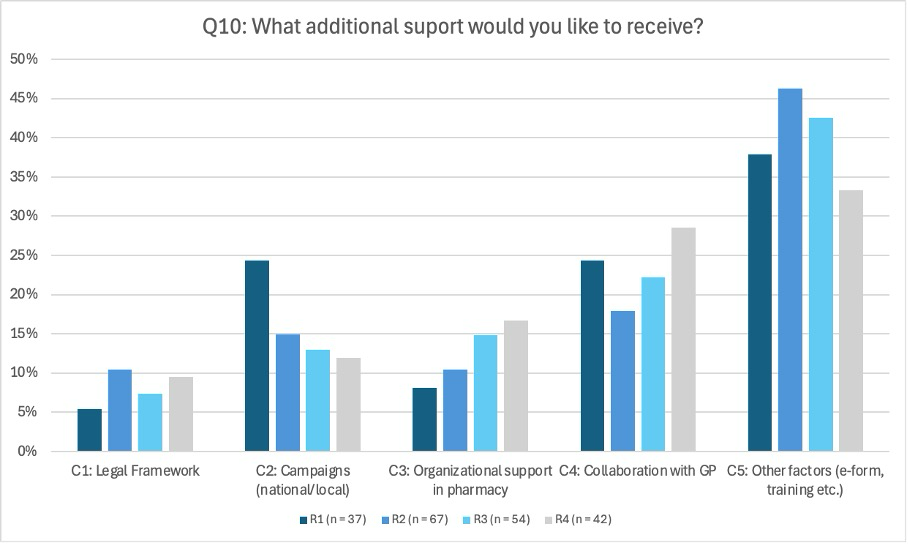

Supplement: Supplementary file 1 [file Supplementaryfile1.docx]
